# Influenza virus decreases albumin uptake and megalin expression in alveolar epithelial cells

**DOI:** 10.3389/fimmu.2023.1260973

**Published:** 2023-09-01

**Authors:** Andrés Alberro-Brage, Vitalii Kryvenko, Christina Malainou, Stefan Günther, Rory E. Morty, Werner Seeger, Susanne Herold, Christos Samakovlis, István Vadász

**Affiliations:** ^1^ Department of Internal Medicine, Justus Liebig University, Universities of Giessen and Marburg Lung Center (UGMLC), Giessen, Germany; ^2^ German Center for Lung Research (DZL), Giessen, Germany; ^3^ The Cardio-Pulmonary Institute (CPI), Giessen, Germany; ^4^ Institute for Lung Health (ILH), Giessen, Germany; ^5^ Max Planck Institute for Heart and Lung Research, Bad Nauheim, Germany; ^6^ Department of Translational Pulmonology, Heidelberg University Hospital, Heidelberg, Germany; ^7^ Science for Life Laboratory, Department of Molecular Biosciences, The Wenner-Gren Institute, Stockholm University, Stockholm, Sweden

**Keywords:** influenza virus, albumin, epithelial cells, lungs, endocytosis

## Abstract

**Introduction:**

Acute respiratory distress syndrome (ARDS) is a common complication of influenza virus (IV) infection. During ARDS, alveolar protein concentrations often reach 40-90% of plasma levels, causing severe impairment of gas exchange and promoting deleterious alveolar remodeling. Protein clearance from the alveolar space is at least in part facilitated by the multi-ligand receptor megalin through clathrin-mediated endocytosis.

**Methods:**

To investigate whether IV infection impairs alveolar protein clearance, we examined albumin uptake and megalin expression in MLE-12 cells and alveolar epithelial cells (AEC) from murine precision-cut lung slices (PCLS) and in vivo, under IV infection conditions by flow cytometry and western blot. Transcriptional levels from AEC and broncho-alveolar lavage (BAL) cells were analyzed in an in-vivo mouse model by RNAseq.

**Results:**

IV significantly downregulated albumin uptake, independently of activation of the TGF-β1/GSK3β axis that has been previously implicated in the regulation of megalin function. Decreased plasma membrane abundance, total protein levels, and mRNA expression of megalin were associated with this phenotype. In IV-infected mice, we identified a significant upregulation of matrix metalloproteinase (MMP)-14 in BAL fluid cells. Furthermore, the inhibition of this protease partially recovered total megalin levels and albumin uptake.

**Discussion:**

Our results suggest that the previously described MMP-driven shedding mechanisms are potentially involved in downregulation of megalin cell surface abundance and clearance of excess alveolar protein. As lower alveolar edema protein concentrations are associated with better outcomes in respiratory failure, our findings highlight the therapeutic potential of a timely MMP inhibition in the treatment of IV-induced ARDS.

## Introduction

Acute respiratory distress syndrome (ARDS) is a life-threatening condition first observed and described in the 1960s as the clinical presentation of acute hypoxemia due to pulmonary gas exchange failure in critically ill adults and children (1). ARDS is a complex syndrome, it cannot currently be diagnosed through laboratory tests, imaging, or any other “gold standard” techniques, and current treatments are based solely on supportive care and mechanical ventilation. More than 10% of all ICU admissions worldwide are due to ARDS cases, and the mortality rate in hospitalized patients is around 30-46% (2). Non-survivors of ARDS exhibit threefold higher levels of precipitated protein in their edema fluid than survivors of the disease, and patients who die of ARDS have large quantities of insoluble protein in their air spaces (3, 4). Therefore, clearance of excess protein from the alveolar space is a both physiologically and clinically important process (2, 5).

Lower respiratory tract infections are a major cause of mortality globally, accounting for 3.2 million deaths worldwide in 2015 (6, 7). Pneumonia, an acute respiratory infection affecting the lungs, can be caused by a variety of pathogens such as respiratory viruses, bacteria, fungi, or their combinations (8–11), and is one of the leading clinical risks that can trigger ARDS. Prior to the coronavirus disease (COVID-19) pandemic, studies investigating adults admitted to the intensive care unit with respiratory failure have reported that respiratory viruses are responsible for 26 to 37% of severe pneumonia cases (12). The prevalence of different viruses identified in patients with lower respiratory tract infections varies depending on the location and season of the study, but the most commonly detected viruses include influenza virus (IV), rhinovirus, coronavirus, respiratory syncytial virus (RSV), human metapneumovirus (hMPV), parainfluenzavirus (PIV), and adenovirus (13).

Protein uptake in the alveolar epithelium is an active, receptor-mediated process. Previous studies by our group have demonstrated that megalin, an endocytic receptor, plays a key role in the uptake of albumin from the alveolar space, and that albumin clearance is significantly reduced in acute lung injury (ALI) (14–17). In ARDS, transforming growth factor-β1 (TGF-β1) is a key cytokine that is able to cause the decrease of protein transport by stimulating the de-phosphorylation and thus activation of glycogen synthase kinase 3β (GSK3β) via protein phosphatase 1 (PP1). Active GSK3β phosphorylates the PPPSP motif located on megalin’s cytoplasmic domain, leading to continuous internalization of the receptor. This negatively regulates megalin recycling and cell surface availability, ultimately resulting in impaired protein uptake (16).

There is an increasing body of evidence indicating that megalin undergoes regulated intramembrane proteolysis (RIP) in response to ligand binding through a Notch-like signaling pathway (17, 18). This process of RIP is conserved and links the function of receptors to intracellular signaling (19, 20). Specifically, it involves the shedding of megalin’s ectodomain by a matrix metalloprotease (MMP), which leads to the production of a distinct membrane-associated carboxyl-terminal fragment (MCTF). Subsequently, the activity of γ-secretase mediates the intramembrane proteolysis of MCTF, which generates a soluble COOH-terminal cytosolic domain that translocates into the nucleus, where it regulates gene expression (17, 18, 21, 22).

MMPs are enzymes produced by various types of cells (stromal, epithelial, and inflammatory) and are capable of degrading all components of the extracellular matrix (ECM). Their upregulation has been observed in broncho-alveolar lavage (BAL) fluid samples of ARDS patients and is believed to contribute to the remodeling of the ECM and the repair of the alveolar-capillary barrier (23, 24). Our group has previously shown that TGF-β1 treatment increases the MMP-2, -9, and -14-mediated shedding and RIP of megalin at the cell surface, resulting in a decrease in the receptor’s abundance and transcriptional downregulation. Silencing of MMP-2, -9, or -14 prevented TGF-β1-induced reduction of megalin cell surface abundance and restored albumin binding and uptake in rat lung epithelial cells (17). This suggested a second mechanism of megalin downregulation by TGF-β1.

In this work, we studied the effects of IV infection on megalin cell surface expression, on the protein uptake process within the alveolar epithelium, and on MMP expression, and its effects on megalin and protein uptake downregulation. Moreover, we show that the use of MMP inhibitors might provide an effective way to reverse those effects.

## Methods

### Cell culture

MLE-12 cells (ATCC CRL-2110), were incubated at 37°C in air containing 5% carbon dioxide and 80-90% humidity. Nutrients were provided in DMEM/F-12, GlutaMAX culture medium containing 2% fetal bovine serum (FBS) solution, and 1% penicillin/streptomycin antibiotics. The culture medium was replaced every two days for cell culture maintenance. All media, supplements, and reactants were purchased from Gibco, ThermoFisher Scientific, Waltham, MA, USA.

### Mouse strains

Animal experiments were performed in wild-type C57BL/6 mice. Precision-cut lung slices (PCLS) were also obtained from wild-type C57BL/6 mice.

### Precision-cut lung slices and *in vitro* culture

The methodology involved in preparing and culturing PCLS has been described elsewhere (25). During experiments, each slice was cultured in 400 µl of culture medium with daily medium changes.

### PCLS tissue dissociation

After treatment, PCLS were dissociated following a modified protocol for AEC isolation from lung tissue (26). Briefly, five PCLS per condition were pooled together and digested for 20 minutes at 37°C with 500 µL of a solution containing elastase 250 ng/mL (EC134, Elastin Products Co. Inc., Owensville, MO, USA) Trypsin/EDTA 0.5% (Gibco, 15400054) in FBS-free DMEM:F12 medium (Gibco, 31331093) with gentle shaking. Cells were then washed in MACS buffer pelleted at 300 x g for 10 min at 37°C, resuspended in PBS and kept on ice for further analysis.

### Virus strain

In all viral infection experiments, the influenza virus (IV) strain used was A/Puerto Rico/8/1934 H1N1 seasonal, mouse-adapted originated by Prof. Stephan Pleschka (Institute of Medical Virology, Justus Liebig University Giessen) and propagated in MDCK II cells.

### Infection with influenza A virus

For IV infection in cell culture, cells were inoculated as previously described (27). Briefly, IV diluted in 0.2%) BSA was added to MLE-12 cells in multiplicities of infection (MOI) of 0.1 or 1. Cells were incubated at 37°C for one hour and then washed once with PBS and incubated with fresh DMEM:F12 0.1% BSA for the required time until the experimental readout. TPCK-treated trypsin was not added into cell cultures as it exhibited toxicity and increased cell death.

### Inhibition experiments

For inhibition studies, the following compounds were used: MT1-MMP inhibitor, NSC405020 (444295 Sigma-Aldrich, Darmstadt, Germany) in 0.1% DMEM, tideglusib SML0339 (Sigma-Aldrich, Darmstadt, Germany). In all cases, compounds were dissolved in DMSO. MLE-12 cells or PCLS were incubated in the presence of the inhibitor or vehicle only in the case of controls.

### *In vivo* experiments

All animal studies were performed according to protocols approved by the Animal Ethics Committee of the Regierungspraesidium Giessen (permit numbers: G71/2018 and GI 20/10 No. 21/2017 No.838-GP). Eight-week-old wild-type C57BL/6 mice were obtained from Charles River Laboratories. Two groups of 5 individuals were created: control and infected. Mice were inoculated intratracheally with 0.35x10^3^ plaque forming units (pfu) of PR8 IV diluted in 70 µL of sterile PBS or 70 µL of sterile PBS alone.

### Tissue sampling and AEC isolation

Samples were harvested 5 days after inoculation. BAL war performed through the intratracheal application of PBS-EDTA, leading to the acquisition of a total volume of 1.2 mL BAL fluid. The superior lobe was fixed in paraformaldehyde (PFA) 4% for 24h at 4°C. The rest of the lung tissue was dissected in DMEM/2.5% HEPES plus 0.01% DNase using the gentleMACS Dissociator (Miltenyi Biotec, Bergisch Gladbach, Germany). Cells were filtered, washed, and resuspended in DMEM, followed by incubation with biotinylated anti-mouse CD31, CD16/32, and CD45. Bound cells were removed by separation with streptavidin magnetic beads. The remaining cells were used for mRNA and protein expression analysis.

### Flow cytometry analysis of immune cell populations in the broncho-alveolar lavage fluid

Multicolor flow cytometry (FC) analysis was performed with an LSR Fortessa using DIVA software (BD Biosciences, NJ, USA). In summary, BAL fluid samples were centrifuged at 1600 rpm, 8 min, 4°C, and resuspended in FACS buffer (PBS, 7.4% EDTA, 0.5% FCS pH 7.2, 0.01% NaAz) containing immune globulin blocking solution [10% Gamunex-C (Grifols, ES)/1% BSA/0.02% NaAz]. Cells were incubated in an antibody mixture for 20 min at 4°C, washed, and resuspended in 400µl FACS buffer. The antibodies used for staining and the dead cell exclusion reagent (7-AAD) were purchased from BioLegend, San Diego, CA, USA. The antibodies were: CD45 APC/Cy7 (10311), Ly6G APC (127614), CD11c PE/Cy7 (117318), CD11b FITC (101205), 7AAD (420403). The antibody against SiglecF BV421 was purchased from BD Biosciences, 565934.Data analysis was performed with FlowJo™ v10.7 software (BD Biosciences, NJ, USA).

### Total protein extraction and quantification

For MLE-12 experiments, cells were washed twice with PBS, and mRIPA buffer (50 mM Tris-HCl, pH 8, 150 mM NaCl, 1% NP-40 (Sigma-Aldrich, Darmstadt, Germany), 1% sodium deoxycholate (Sigma-Aldrich, Darmstadt, Germany), cOmplete Protease Inhibitor Cocktail (Roche) was added to the cell cultures and incubated on ice for 10min for protein extraction. In the case of PCLS, after treatment, five PCLS were pooled together and incubated in mRIPA buffer for 10 min on ice. PCLS were then mechanically disrupted using a tissue homogenizer (Polytron 1200E, Kinematica AG, Malters, Switzerland) and then centrifuged at 4°C for 10 min at 10,000 x g. Protein concentrations of the supernatants of disrupted cells, PCLS, or BAL fluid were measured by Bradford assay (BioRad, Hercules, CA, USA) according to manufacturer recommendations. Measurements of absorbance were performed in a spectrophotometer (Mo. 6131, Eppendorf, Hamburg, Germany).

### SDS PAGE and western blotting

In order to achieve a better resolution of a wide range of molecular proteins, a TRIS/Acetate SDS-PAGE system (28) was optimized to analyze protein expression. Briefly, samples were denaturalized with LDS sample buffer (NP0007, Invitrogen, ThermoFisher Scientific, Darmstadt, Germany) for 10 min at 70°C. Gradient gels from 3 to 10% of poly-acrylamide (Carl Roth, Karlsruhe, Germany) were prepared, and equal amounts of protein were loaded for each sample. After separation, the proteins were transferred to PVDF membranes (Amersham Hybond P 0.45 µm, GE Healthcare Life Sciences) for 1.5 hours at a fixed intensity of 200 mA in transfer buffer (1% methanol, 0.01% SDS, 25 mM Bicine, 25 mM Bis-Tris, 1 mM EDTA, 1.3 mM sodium bisulfite pH 7.2, Carl Roth, Karlsruhe, Germany) and blocked for 1 hour in 5% nonfat-dried bovine milk (M7409 Sigma-Aldrich, Darmstadt, Germany) T-TBS buffer. Membranes were then incubated overnight with specific antibodies at 4°C. Primary antibodies were washed in T-TBS for 15 minutes and incubated for 1 hour at RT with secondary antibodies conjugated to horseradish peroxidase (HRP). The antibodies used were: Megalin (Proteintech, San Diego, CA, USA), Phospho-GSK-3α/β(Ser21/9) (Cell Signaling, Danvers, MA, USA), GSK-3β (Cell Signaling, Danvers, MA, USA), Phospho-SMAD2 (Ser465/Ser467) (Cell Signaling, Danvers, MA, USA), Smad-2 (Cell Signaling, Danvers, MA, USA), Influenza A NP (Santa Cruz Bio., Dallas, TX, USA), β-Actin (Santa Cruz Bio., Dallas, TX, USA), MMP-14 (ThermoFisher), MMP-2 (Santa Cruz Bio., Dallas, TX, USA), MMP-9 (Santa Cruz Bio., Dallas, TX, USA), MMP-19 (Novus Bio., Littleton, CO, USA), Transferrin receptor (Invitrogen, ThermoFisher Scientific, Darmstadt, Germany), Anti-rabbit IgG HRP-linked (Cell Signaling, Danvers, MA, USA), Anti-mouse IgG HRP-linked (ThermoFisher Scientific, Darmstadt, Germany), Transferrin receptor (Invitrogen, ThermoFisher Scientific, Darmstadt, Germany). Membranes were developed with SuperSignal West Pico or Femto Chemiluminescent Substrate detection kit (Thermo Scientific, Waltham, MA, USA), in a CP 1000 automatic film processor (AGFA, Mortsel, Belgium). Densitometric quantification of bands was made using ImageJ software (National Institutes of Health, Bethesda, Maryland, USA).

### Surface proteins biotinylation

MLE cells were plated in 30 mm Petri dishes 24 h prior to viral infection or mock treatment. After treatment, cell surface proteins were pulled down as previously described (17), after which SDS PAGE and western blot in a Tris/Acetate system were performed.

### Enzyme-linked immunosorbent assay

Mouse TGF-β1 ELISA Kit (Colorimetric, NBP1-92671 Novus Biologicals, Wiesbaden, Germany) was used according to the manufacturer’s instructions to determine concentrations of active and total TGF-β1 released from samples into the culture media or bronchoalveolar lavages. Colorimetric detection was performed in a microplate reader (Infinite 200, Tecan Group, Maennedorf, Switzerland).

### Flow cytometric analysis of albumin uptake

For flow cytometry analysis of labeled albumin uptake, MLE-12 cells or PCLS were treated according to the experimental design described above and incubated at 37°C or 4°C with a solution 50 μg/mL (MLE-12 cells) or 100 μg/mL (PCLS) of albumin from bovine serum (BSA) conjugated with AlexaFluor 488 (A13100 Invitrogen, ThermoFisher Scientific, Darmstadt, Germany). After incubation, MLE-12 cells were washed with PBS and treated with ice-cold Solution X for 10 min, recovered in microcentrifuge tubes, pelleted at 300 x g for 10 min at 4°C, washed in PBS+/+ and stained for 15 min at RT with Zombie Violet Fixable Viability Kit (which has an excitation/emission spectrum similar to the PacificBlue fluorophore) (423114, BioLegend, San Diego, CA, USA), 1/500 dilution before being analyzed by flow cytometry. For PCLS, samples were processed as previously described (29).

### H&E stainings

Lung samples were fixed in formaldehyde 4% at 4°C for 24 h, then washed with PBS, embedded in paraffin, sectioned at 5 μm thickness in a microtome (Leica Biosystems, Wetzlar, Germany), and fixed in glass slides. Hematoxylin/Eosin staining was performed according to standard procedures.

### Image acquisition and analysis

For mean linear intercept (MLI) calculations, hematoxylin-eosin-stained paraffin sections were imaged with a bright field Axioimager microscope (Zeiss, Oberkochen, Germany) at 40X magnification. Measurements of average wall thickness, airspace percentage, and mean linear intercept were made with Leica Qwin Image Processing and Analysis Software.

### RNA extraction

Treated MLE-12 cells, as well as primary AECs-enriched samples and BAL cells, were processed for RNA extraction using the RNeasy Mini Kit (Qiagen, Hilden, Germany) according to the manufacturer’s instructions. The extraction was followed by on-column DNAse digestion (RNase-Free DNase Set, Qiagen, Hilden, Germany).

### Bulk mRNA analysis

RNA integrity was verified by Bioanalyzer RNA 6000 Nano assays (Agilent Technologies, Santa Clara, CA, USA). Library preparation and sequencing were performed in the Deep Sequencing Platform of the Max Planck Institute for Heart and Lung Research, (Bad Nauheim, Germany) by Dr. Stefan Günther. Library preparation integrity was verified with LabChip Gx Touch 24 (Perkin Elmer). For BAL fluid cells, 10 ng of total RNA was used as input for SMARTer^®^ Stranded Total RNA-Seq Kit - Pico Input Mammalian (Takara Bio). For AECs, 100 ng of total RNA was used as input for SMARTer Stranded Total RNA Sample Prep Kit - HI Mammalian (Takara Bio). Sequencing was performed on the NextSeq500 instrument (Illumina) using v2 chemistry, resulting in an average of 35M reads (BAL) or 27M reads (AECs) per library, with a 1x75bp single end setup. The resulting raw reads were assessed for quality, adapter content and duplication rates with FastQC (Andrews S., FastQC: a quality control tool for high throughput sequence data. Avail. at http://www.bioinformatics.babraham.ac.uk/projects/fastqc). Trimmomatic version 0.39 was employed to trim reads after a quality drop below a mean of Q20 in a window of 10 nucleotides (30). Reads between 30 and 150 nucleotides were cleared for further analyses. Trimmed and filtered reads were aligned versus the Ensembl mouse genome version mm10 (GRCm38) using STAR 2.6.1d with the parameter “–outFilterMismatchNoverLmax 0.1” to increase the maximum ratio of mismatches to mapped length to 10% (31) The number of reads aligning to genes were counted using the featureCounts 1.6.5 tool from the Subread package (32). The reads that were admitted and aggregated per gene were the ones that mapped at least partially inside exons. Reads that were overlapping multiple genes or aligning to multiple regions were excluded. DESeq2 version 1.18.1 was used to identify differentially expressed genes (33). Only genes with a minimum fold change of +- 1.5 (log2 +-0.59), a maximum Benjamini-Hochberg corrected p-value of 0.05, and a minimum combined mean of 5 reads were considered as significantly differentially expressed. The Ensemble annotation was enriched with UniProt data (release 06.06.2014) based on Ensembl gene identifiers (34). Transcripts per million (TPM) were calculated as described by Wagner et al. (35)

### cDNA synthesis

RNA extracted from the treated MLE12 cell cultures was used as a template for cDNA synthesis. iScript cDNA synthesis kit (Bio-Rad, Hercules, CA, USA), according to the manufacturer’s instructions.

### Quantitative real-time polymerase chain reaction

qRT-PCR technique was used to quantify the levels of expression of mRNA in the samples. The kit iTaq Universal SYBR Green Supermix (BioRad, Hercules, CA, USA) was used according to the manufacturer’s protocol and the mouse-specific primers combinations used were: LRP2 FW: 5’ AGG CCA CCA GTT CAC TTG CT 3’, LRP2 RV: 5’ AGG ACA CGC CCA TTC TCT TG 3’, TGF-β1 FW: 5’ TGG AGC AAC ATG TGG AAC TC 3’, TGF-β1 RV: 5’ GTC AGC AGC CGG TTA CCA 3’, 18S FW: 5’ AGT CCC TGC CCT TTG TAC ACA 3’, 18S RV. 5’ GAT CCG AGG GCC TCA CTA AAC 3’. Ribosomal 18S served as a normalization control. Data are presented as ΔCt (Ct_target gene_ –Ct_reference gene_).

### Specific mRNA knockdown

Megalin knockdown was performed with small interference RNA technology (Lipofectamine RNAiMAX, ThermoFisher Scientific, Waltham, MA, USA) in OptiMEM medium (Gibco), as previously described (17).

### Statistical analysis

All data is given as mean ± standard deviation (SD). Statistical significance between two groups was analyzed by unpaired Student’s t-test, while differences between three or more groups were analyzed using randomized-block-experiments, one-way ANOVA, and Dunnett’s multiple comparisons test (GraphPad Prism v.5 and v.8). Differences were considered significant when p-values were inferior to 0.05. *p<0.05; **p<0.01; ***p<0.005; ****p<0.001.

## Results

### Influenza virus downregulates albumin uptake in epithelial cells

We set out to analyze the infective capability of the PR8 mouse-adapted influenza A virus strain in MLE-12 cells. In order to quantify the infection levels and cell death rates, we analyzed infected MLE-12 cells by FC (**Supplementary Figures 1A–C**). To detect the presence of the virus, we used an antibody against influenza A H1N1, which allows the detection of the viral protein HA once it is expressed by the cells. We found that the percentage of infected cells correlates with the MOI and the incubation time, as do cellular death rates (**Supplementary Figure 1B**), which demonstrates that the virus can infect MLE-12 cells in culture. We also infected MLE-12 cells at MOIs 0.1 and 1, and after 24h of infection, the cells were lysed and analyzed by western blot against influenza nucleoprotein (NP) (**Supplementary Figure 1D**). The NP signal intensity correlates with the MOI, being stronger at MOI 1 when compared with MOI 0.1.

**Figure 1:**
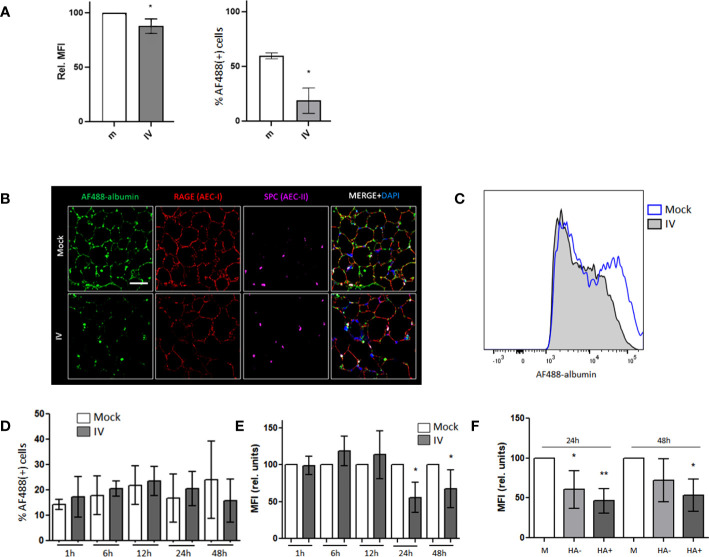
*Albumin endocytosis is impaired in IV-infected AECs.* **(A)** MLE-12 cells were inoculated with IV for 24 h and incubated with AF488-albumin for 1 h before being analyzed by FC. Image shows the relative median fluorescence intensity (MFI) of AF488 (left) and the percentages of cells that stained positive for AF488 (right). **(B)** Confocal images of PCLS mock and IV inoculated with 1x10^6^ pfu of IV for 24 h, incubated in AF488-albumin (green) 250 µg/mL for 60 min and then fixed and stained with RAGE (red) and SPC (purple) antibodies. Scale bar 50 µm. Zoom 63X. **(C–F)** PCLS were inoculated with IV for 1 h, 6 h, 12 h, 24 h and 48 h and incubated in AF488-albumin for 60 min. Images show FC analysis of albumin uptake in EpCAM+ cells. **(C)** Representative histograms for AF488-albumin fluorescence in AEC from mock and IV inoculated PCLS at 24h post inoculation. **(D)** Percentage of AF488-albumin+ cells. **(E)** AF488 MFI in the general AEC population from mock and IV-inoculated PCLS. **(F)** Graph showing the MFI analysis from AEC from mock PCLS (M), AEC from inoculated PCLS that did not express (HA-) or that expressed viral proteins (HA+). All bar graphs show mean ± SD of 3 to 6 independent experiments. Statistic comparisons are relative to mock controls. *p<0.05; **p<0.01.

In order to study the effect of IV infection on albumin uptake in MLE-12 cells, we inoculated the cultured cells with IV at a multiplicity of infection (MOI) of 1 and control (mock infection) for 24 h. After incubation with AF488-albumin, we analyzed fluorescence levels in control and infected MLE-12 cells by FC (**Figure 1A**). We found a 15% decrease in albumin uptake in the infected cells compared to control, as depicted by the lower relative MFI, as well as a reduction in the percentage of cells staining positive for albumin from 59.6% to 18.7%.

We have previously reported that albumin uptake can be reliably studied in PCLS (29). This model is more representative of the lung environment than a cell monoculture and our next step was to characterize the viral infection in PCLS. To assess the infectious capability of the mouse-adapted PR8 IV strain in PCLS, we added 1x10^6^ pfu to the culture medium per slice of tissue, incubated for 12, 24, or 48 h before fixation, and stained with an influenza nucleoprotein (NP) specific antibody. Confocal microscopy imaging results showed the presence of NP in all three time points, with the highest expression observed after 24 and 48h of infection (**Supplementary Figure 1E**). By FC, we were able to quantify the infection levels in alveolar epithelial cells (AEC) at 1, 6, 12, 24, and 48 h after infection (**Supplementary Figure 1F**) and found that, after 24 and 48 h, between 20 and 30% of cells were infected, thus, we decided to incubate with the virus for 24h as time-frame for all PCLS experiments.

In order to measure albumin uptake levels in AECs of PCLS, we inoculated them with IV for 24 h and incubated them in AF488-albumin for 60 min. By using confocal microscopy, we were able to detect a co-localization of the AF488-albumin signal with the receptor for advanced glycation end products (RAGE) and surfactant protein C (SPC), suggesting that albumin is being taken up by both AT1 and AT2 cell types (36–38)(**Figure 1B**). Remarkably, the AF488 signal was substantially decreased in the IV infected PCLS (**Figure 1B**). When we compared the levels of albumin uptake in AEC from mock and IV- inoculated PCLS by FC analysis (**Figures 1C–F**), we found that the percentage of cells that internalized albumin did not vary significantly (**Figure 1D**). However, upon analyzing the relative median fluorescence intensity (MFI), we observed a marked reduction in the AF488-albumin signal in AEC from the IV-inoculated samples after 24 h and 48 h post-inoculation (**Figure 1E**). Furthermore, when analyzing the AEC from inoculated slices and distinguishing between infected (HA+) and non-infected (HA-) cells, we found that the decrease in albumin uptake levels, measured as MFI, persisted in both infected and non-infected cells (**Figure 1F**).

### Influenza virus induces downregulation of megalin expression at the transcriptional level

**Figure 2:**
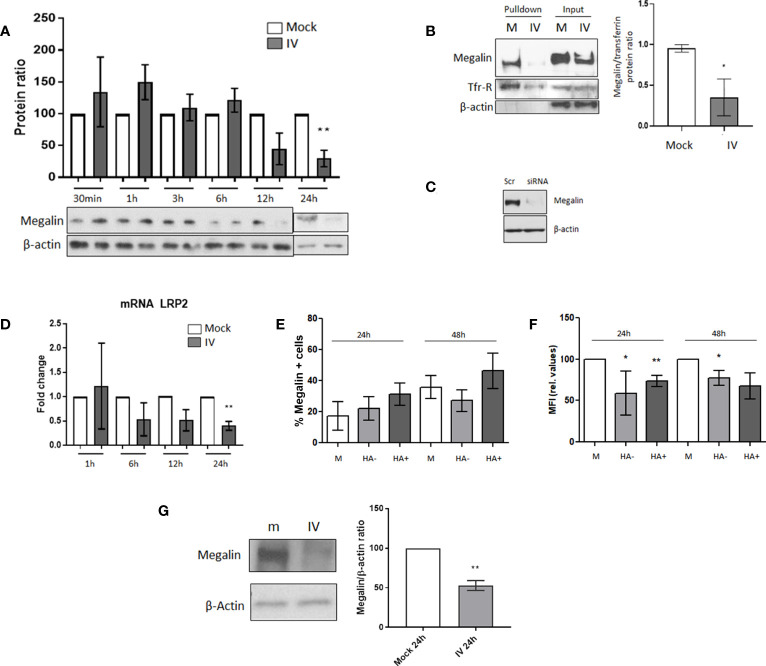
*Megalin cell surface expression is downregulated in AECs under IV infection conditions.* **(A)** MLE-12 cells were inoculated with IV at MOI 1, incubated for 30 min, 1 h, 3 h, 6 h, 12 h and 24 h The graph shows the western blot densitometry analysis normalized to beta-actin and to each mock control. A representative blot for each time point is shown. **(B)** Cell-surface biotinylation assay was performed in MLE-12 cells inoculated with IV MOI 1 incubated for 24 h Left: representative blot. Right: Densitometry analysis and quantification of megalin, normalized to transferrin. **(C)** Antibody specificity characterization: megalin siRNA was transfected into to MLE-12 cells and megalin expression was analyzed by western blot. Scr: scrambled RNA (control). **(D)** mRNA was isolated from mock and IV-treated cells, at 1, 6, 12 and 24 h post infection. LRP2 transcription levels were quantified by qPCR. **(E, F)**. PCLS were treated in mock or IV conditions. After dissociation, live AEC were analyzed by FC. **(E)** Percentages of cells expressing megalin on their surface at 24 h and 48 h post inoculation. EpCAM+ cells from mock PCLS (M) or from IV-inoculated PCLS expressing (HA+) or not expressing (HA-) viral proteins are shown. **(F)**: Relative MFI for megalin expression on the cell surface, calculated as MFI APC of cells stained with an APC-megalin antibody- MFI of FMO control. **(G)** PCLS mock (m) and IV-inoculated (IV) were incubated for 24 h and extracted proteins were analyzed by western blot. Left: Representative immunoblot for total cell megalin expression. Right: Densitometric quantification of megalin (600 kDa) expression, relative to actin and mock. All bar graphs show mean ± SD of 3 to 6 independent experiments. Statistic comparisons are relative to mock controls. *p<0.05; **p<0.01.

Our previous studies have shown that megalin plays a crucial role in albumin endocytosis in the alveolar epithelium (14, 16). Therefore, we conducted experiments to investigate the expression of megalin on infected AECs. We inoculated MLE-12 cells with IV at MOI 1 and incubated them for different time periods of 30 min, 1 h, 3 h, 6 h, 12 h, and 24 h. We then analyzed the expression of megalin by western blot (**Figure 2A**). Results indicated a significant reduction in total megalin expression after 24 h of incubation, with an average decrease of ~70%. To determine whether this decrease was reflected in the levels of the endocytic receptor located on the cell surface (as opposed to intracellular compartments), we performed a cell-surface protein biotinylation assay on the inoculated cells after 24 h of incubation (**Figure 2B**). The densitometric quantification of the results showed an average reduction of the receptor on the plasma membrane of approx. 63%, compared to the control. The specificity of the megalin antibody was validated by small interfering RNA knockout of megalin in MLE-12 cells (**Figure 2C**).

To further investigate whether the decrease in megalin expression was due to downregulation at the transcriptional level, we isolated RNA from control and IV-treated cells at different time points of 1, 6, 12, and 24 h after inoculation. We then quantified the levels of LRP2 (the megalin gene) by qPCR (**Figure 2D**). The results revealed a decreasing trend in LRP2 transcription levels at the 6 h time point and a significant downregulation at 24 h post-inoculation.

To investigate megalin expression levels in AEC under IV infection conditions, we conducted flow cytometry analysis. Specifically, we measured megalin cell surface expression in AEC from mock and IV-inoculated PCLS after 24 and 48 h (**Figures 2E, F**). We used a specific megalin antibody and only live cells were analyzed to ensure that only cell surface megalin was detected (**Supplementary Figure 1C**). Our results showed no significant differences in the percentage of cells expressing megalin (**Figure 2E**). However, we did find a significant decrease in the median fluorescence intensity (MFI) of megalin in AEC from inoculated samples (**Figure 2F**), both at 24 and 48 h, in both infected (HA+) and non-infected (HA-) cells. These results reflect the reduced number of receptors on the cell surface. Moreover, western blot analysis of the total amount of megalin in PCLS homogenates revealed a substantial decrease in the full-length megalin abundance in the inoculated samples after 24 h of IV infection (**Figure 2G**).

### Decrease of megalin expression is independent of the TGF-β/GSK3β axis

**Figure 3:**
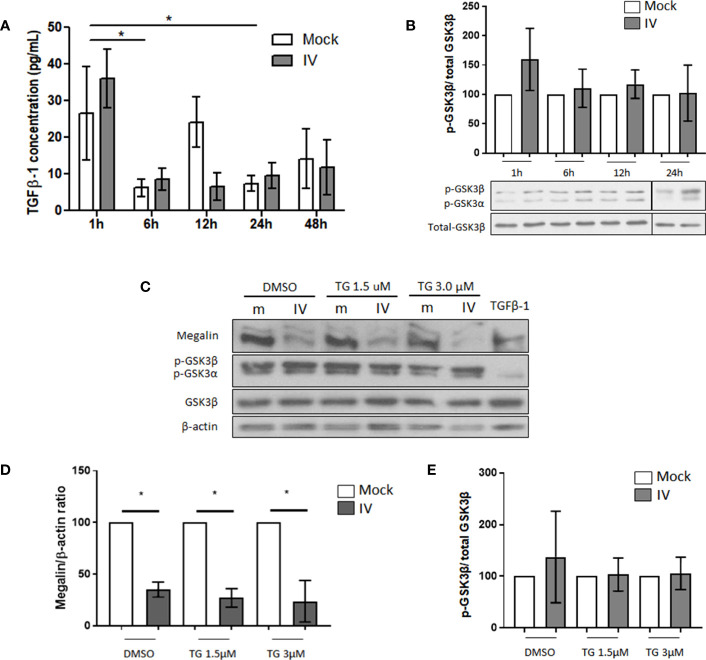
*Downregulation of megalin in IV infected PCLS is independent of GSK3β activation.* **(A)** Quantification of active TGF-β1 in media from mock and IV-inoculated PCLS incubated for 1 h, 6 h, 12 h, 24 h and 48 h **(B)** Phosphorylation levels of GSK3β in PCLS homogenates, at 1 h, 6 h, 12 h and 24 h post IV inoculation. **(C–E)** PCLS were treated with the irreversible GSK3β inhibitor tideglusib (TG) at two different concentrations (1.5 µM and 3 µM) or with a vehicle control (DMSO) for 24 h prior to inoculation with IV. Proteins were extracted and analyzed by western blot. **(C)** Representative immunoblots for megalin and phospho-GSK3β. TGF-β1 20 ng/mL was used as a TGF-β1 activity positive control. **(D)** Densitometric quantification of megalin in C. **(E)** Densitometric quantification of phospho-GSK3β in **(C)** All graphs show mean ± SD of 3 to 6 independent experiments. *p<0.05.

Our previous research had indicated the existence of at least two pathways for megalin downregulation in alveolar epithelial cells. One pathway involves promoting megalin internalization from the cell surface through the TGF-β1/GSK3β axis and the second pathway involves the shedding of megalin through MMP -2, -9, and -14. To investigate whether the TGF-β1/GSK3β/megalin axis plays a role in downregulation of megalin expression in this *ex vivo* model, we conducted a time-course analysis to measure the concentration of active TGF-β1 in the media of mock and IV-inoculated PCLS (**Figure 3A**) and MLE-12 cells (**Supplementary Figure 2A**) using ELISA. We did not observe any significant differences in the concentration of active TGF-β1 between mock and infected PCLS for any time points, despite some variation in the levels of TGF-β1 over time.

When we analyzed the levels of SMAD2 phosphorylation, a downstream target of the TGF-β1 canonical pathway in IV-treated MLE-12 cells, we found a decrease during the first 3 h and no change when compared to the mock control at 6, 12, and 24 h, suggesting no TGF-β1 activation during the experimental time frame (**Supplementary Figure 2C**). When we analyzed the phosphorylation levels of GSK3β, we found an increasing trend during the experimental time frame (as opposed to de-phosphorylation, which is associated with activation of the kinase), indicating that there was no activation of GSK3β in our setting (**Supplementary Figure 2D**). Additionally, the levels of phosphorylated GSK3β in treated PCLS did not show any activation up to 24 h post-IV inoculation (**Figure 3B**). To investigate whether the inhibition of GSK3β can reverse the downregulation of megalin in infected PCLS, we treated the PCLS with the GSK3β inhibitor tideglusib (TG) at two different concentrations (1.5 and 3.0 µM) for 24h before IV inoculation. Our results showed that the inhibition of GSK3β did not lead to a recovery of whole-length megalin protein levels in infected PCLS (**Figures 3C–E**).

**Figure 4:**
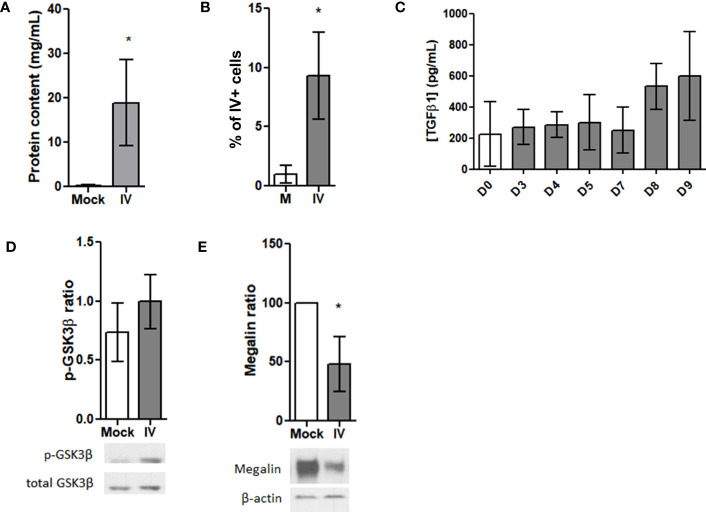
*Megalin is downregulated in AEC from IV-infected mice.* **(A–F)** Lung samples were obtained from mice 5 days after intratrachaeal instillation of 350 pfu of IV (n=5). **(A)** Whole protein contents from the BAL fluid of control (Mock) and infected (IV) mice. **(B)** AEC from lung samples were analyzed by FC for viral proteins (HA). Graph shows percentages for HA+ cells in mock (M) and infected (IV) samples. **(C)** TGF-β1 levels were measured in the BALF from control and IV-infected mice following 3, 4, 5, 7, 8 and 9 days of infection by ELISA. **(D, E)** AEC were isolated and analyzed by western blot. **(D)** Top: Densitometric quantification of p-GSK3β. Bottom: Representative blot. **(E)** Top: Densitometric quantification of megalin. Bottom: Representative blot. *p<0.05.

Since TGF-β1 is produced also by numerous immune cells (39), we performed an *in vivo* experiment using infected mice, and examined TGF-β1 levels, megalin expression, and GSK3β phosphorylation in the infected alveolar epithelium. We infected mice intratracheally with either IV or PBS (mock) and then assessed lung tissues and BAL fluid samples five days following inoculation. The analysis of the hematoxylin/eosin stainings of the alveolar region exhibited a rising trend in the mean thickness of the alveolar wall in the infected mice (**Supplementary Figures 3A, B**). We also saw a significant increase in protein content in the BAL fluid samples (**Figure 4A**), which suggests alveolar barrier leakage. When assessing the levels of infection by FC, we found the AEC infection percentage from the inoculated mice was close to 10% (**Figure 4B**). An analysis of BALF cells revealed that the infected samples had significantly lower resident tissue-resident alveolar macrophages (TRAM) but a higher percentage of polymorphonuclear leukocytes (PMN) and bone marrow-derived macrophages (BMDM) (**Supplementary Figure 3C**), which suggests an active immune response to the IV infection.

We obtained BAL fluid samples from mice infected with IV at various time points including day 0 (control), and days 3, 4, 5, 7, 8, and 9 post-infection and used ELISA to measure active TGF-β1 concentrations. Although no significant differences were observed among the samples, there was an upward trend in the levels of active TGF-β1 from day 8 onwards (**Figure 4C**). Additionally, analysis of AEC protein extracts by western blot showed no activation (no dephosphorylation) of GSK3β (**Figure 4D**). However, an evident decrease in the amount of total, whole length megalin was observed (**Figure 4E**).

**Figure 5:**
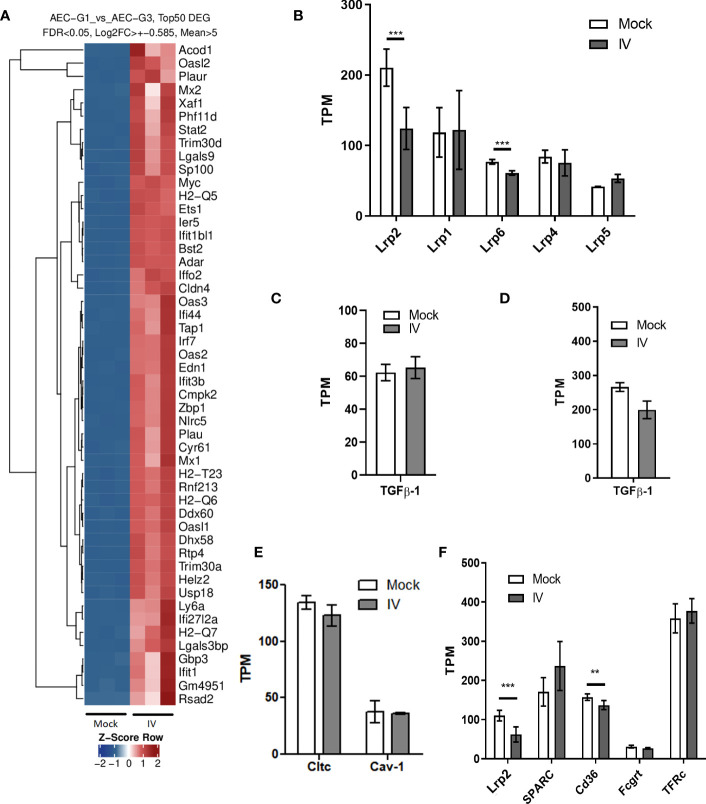
m*RNA expression in AEC and BAL fluid cells from IV-infected mice.* **(A)** Top 50 differentially expressed genes (DEG) in AEC. **(B)** mRNA levels of the top 6 most expressed LDL receptor family members in AEC. **(C, D)** *Tgfb1* mRNA levels in AEC **(C)** and BAL fluid cells **(D)**. **(E)** mRNA levels of clathrin (*Cltc*) and caveolin-1 (*Cav-1*) in AEC. **(F)** mRNA expression levels of the albumin receptors megalin, secreted protein acidic and rich in cysteine (Sparc), the scavenger receptor Cd36, and neonatal Fc receptor (Fcgrt) in mock and infected (IV) AEC. Expression levels or transferrin receptor (TFr) are shown for comparison. Normalized RNAseq data, n=3. Bars represent mean ± SD. TPM: transcripts per million. **p<0.01; ***p<0.005.

We also analyzed the RNA expression profiles from AEC and BAL fluid immune cells, by performing deep sequencing of all mRNA species (RNAseq). We found that the majority of the differentially expressed genes (DEG) in AEC under influenza virus infection conditions are related to innate immune response activation and anti-viral response genes (**Figure 5A**). Members of the LDL receptor-related proteins (LRP) family have been described to undergo RIP via a receptor processor mechanism in response to ligand binding, which in turn results in receptor downregulation (40, 41). We found a marked downregulation of LRP2 (megalin) mRNA expression in the infected samples. We then analyzed if IV treatment triggers downregulation not just in megalin but in other members of the LRP family as well. Interestingly, the data demonstrated that in our experimental conditions, only megalin and Lrp6 showed a significant decrease upon IV infection (**Figure 5B**).

When we looked for TGF-β1 mRNA expression, we found no significant differences either in AEC or BAL fluid cells between mock and infection conditions (**Figures 5C, D**). Of note, neither clathrin nor caveolin-1, proteins involved in the coating of vesicles in cellular endocytosis, showed significant variations in mRNA expression levels (**Figure 5E**). We sought to identify receptor molecules that are known to bind albumin, namely megalin, secreted protein acidic and rich in cysteine (SPARC), the scavenger receptor Cd36, and neonatal Fc receptor (Fcgrt) (42, 43) in AEC. The results showed that only megalin and Cd36 were significantly downregulated in the samples infected with IV. On average, the mRNA counts for megalin decreased by more than 50% (fold change = 0.49), and for CD36 by 24% (fold change = 0.76) (**Figure 5F**).

### MMP expression profiles in the lung under IV infection conditions

**Figure 6:**
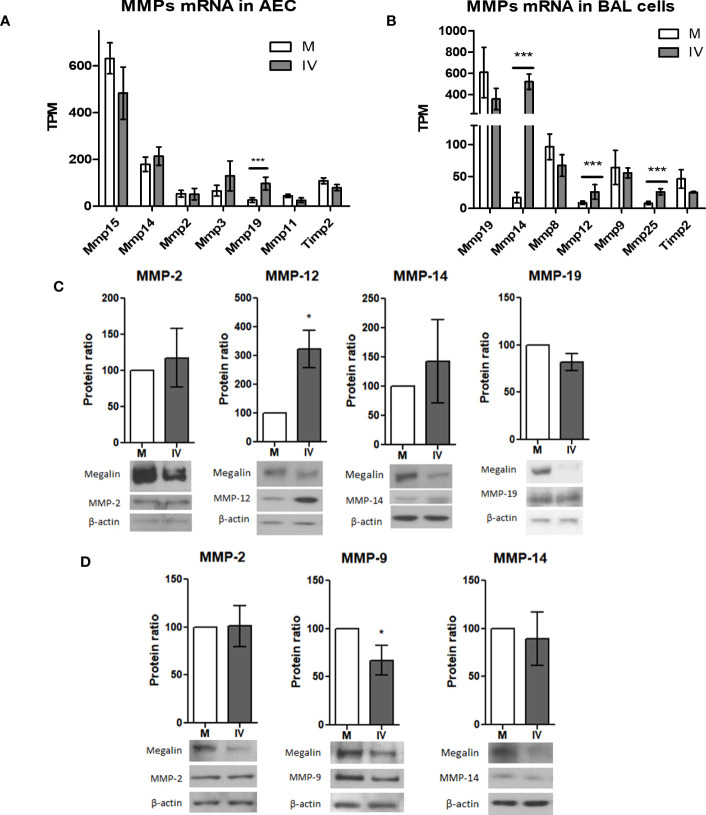
*IV infection alters MMP expression levels in AEC and BAL fluid cells.***(A, B)** mRNA levels of the top 6 most expressed MMP and *Timp2* in AEC **(A)** and BAL **(B)** from infected mice. Normalized RNAseq data, n=3. Bars represent mean ± SD. TPM: transcripts per million. **(C, D)** MLE-12 cells **(C)** or PCLS **(D)** were inoculated with IV for 24 h and the protein extracts analyzed by western blot. Representative blots and MMP/actin densitometry ratio graphs are shown. Values represent mean ± SD, n=3. **(C)** In MLE-12 cells protein expression of MMP-12 was upregulated in IV conditions, while expression of MMP-2, MMP-14 and MMP-19 did not show significant differences. Representative megalin and actin immunoblots are shown. **(D)** In PCLS, protein expression of MMP-9 was downregulated, while no significant differences were found in MMP-2 and MMP-14 expression. *p<0.05; ***p<0.005.

Previously, our group reported that the activity of MMP-2, -9 and -14 plays a role in the shedding and downregulation of megalin expression in alveolar epithelial cells (17). In our *in vivo* RNAseq data, we found that influenza virus infection altered the expression of MMP genes in the alveolar epithelial and BAL fluid cells (**Figures 6A, B**). In AEC from IV-inoculated mice, MMP-19 was the only metalloprotease that showed a significant increment, while the expression of MMP-14 showed a moderate upregulation tendency (**Figure 6A**). In BAL fluid cells, MMP-14 expression was markedly upregulated in IV-infected mice, with an approx. 50-fold change, compared to control. At the same time, expression levels of TIMP-2, the endogenous inhibitor of MT1-MMP (44), were not significantly altered (**Figure 6B**).

Next, we looked for MMP protein expression in MLE-12 cells and PCLS in culture. Western blot analysis of protein expression in IV-inoculated MLE-12 cells showed an upregulation of MMP-12 24h post infection. The expression levels of MMP-2, MMP-14, and MMP-19, were not significantly increased (**Figure 6C**). In IV-inoculated PCLS, expression of MMP-9 was downregulated, while the protein levels of MMP-2 and MMP-14 were unchanged (**Figure 6D**).

### Inhibition of MMP-14 activity rescues megalin expression and albumin uptake in epithelial cells

**Figure 7:**
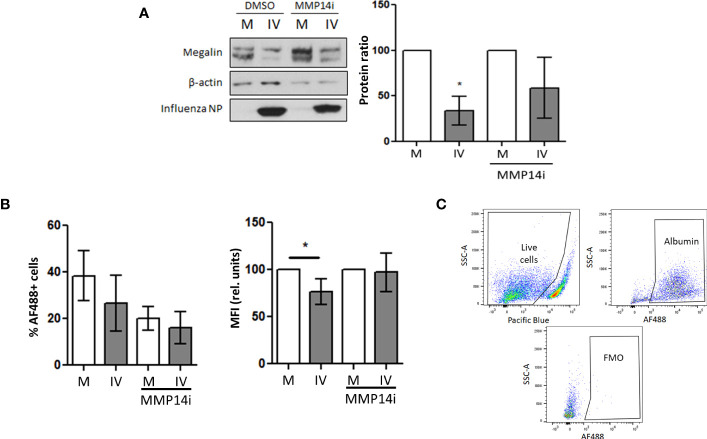
*Inhibition of MMP-14 partially recovers megalin abundance and albumin uptake in AEC.* **(A)** MLE-12 cells were inoculated with IV (MOI 1) and incubated with the specific MMP14 inhibitor NSC405020 200µM (MMP14i) or vehicle (DMSO) for 24 h Total megalin amounts were analyzed by western blot. Left: representative blot. Right: Densitometry quantification normalized to beta actin. **(B)** PCLS were incubated in mock (M) or influenza virus (IV) conditions for 24 h in the presence of MMP14 inhibitor NSC405020 (MMP14i) 200 µM or vehicle (DMSO) and incubated with AF488-albumin for 1 h Left: Flow cytometry data showing the MFI of AF488 in AEC. Right: % of live cells per condition. n=3. **(C)** Gating strategy and FMO control. *p<0.05.

Because MMP-14 has been described to play a role in megalin processing (17) and our *in vivo* results show a significant upregulation of this enzyme in BAL fluid cells, we next analyzed if the downregulation of megalin could be reverted by inhibition of MMP-14 by NSC405020 (a small molecule that specifically inhibits MMP-14 homodimerization via the MMP-14 PEX domain (45)) in MLE-12 cells and PCLS cultures. In MLE-12 cells the presence of the inhibitor in the IV-inoculated samples was able to partially recover the protein expression levels of megalin (**Figure 7A**). Moreover, it partially rescued albumin uptake in PCLS as measured by FC (**Figures 7B, C**).

## Discussion

ARDS accounts for more than 10% of all ICU admissions in the world and has an associated mortality of approximately 40%. Different pathologies can lead to this syndrome, with IV infection being one of the most common causes (13). During ARDS, fluid and protein accumulation in the alveolar space -due to increased alveolar-capillary barrier permeability- impairs gas exchange. Protein concentration in the alveoli is three times higher in non-survivors than in patients who recover from the disease (3). Hence, clearance of excess protein from the alveolar space is critical to improving the outcome of ARDS. However, as of today, no effective targeted therapeutic treatments are available, highlighting an unmet medical need for further scientific approaches.

During ALI and ARDS, repair of the alveolar-capillary barrier in combination with an effective fluid and protein removal are needed to restore alveolar function. Several studies from our group have aimed to understand the underlying mechanisms and pathways that are involved in these processes (14–17, 46)

In the current study, we were able to show that inoculation with Influenza A/Puerto Rico/8/1934 H1N1 strain led to productive infection in MLE-12 cells, PCLS, and an *in vivo* mouse model, and that our infection models proved to be effective tools to study the effects of influenza virus infection on the lung epithelium.

Infected AEC from PCLS showed a marked decrease in albumin uptake levels, which correlated with a decrease in the expression of megalin. Although the number of cells that internalized labeled albumin did not change significantly, the amount of endocytosed albumin was significantly reduced. This is consistent with the idea of a downregulation in the abundance of albumin receptor on the cell surface, which would not affect the percentage of cells that take up albumin, but would, instead, reduce the amount of internalized albumin. Moreover, when we compared cell surface and total megalin levels in mock and IV-inoculated cells, we found a strong downregulation as early as 12 h post infection. These differences occurred not only translationally but also transcriptionally, suggesting that IV induces megalin downregulation at both levels.

Interestingly, in IV-inoculated PCLS, albumin uptake levels were downregulated in both infected (HA+) and non-infected (HA-) cells and the same effect was observed for the cell surface expression of megalin. This suggests that the downregulation of the protein uptake and megalin abundance is independent of the infection status of the cells and that the presence of the virus in the medium is sufficient to downregulate megalin, either through a direct effect or by paracrine cell communication.

In contrast to other ALI settings (14), the decrease in albumin uptake and in megalin expression occurred even when neither TGF-β1 nor GSK3β activities were increased. In fact, when we used the specific GSK3β inhibitor tideglusib, we found no recovery in megalin levels. Collectively, these data suggest that GSK3β activity is not required to drive the downregulation of megalin in our experimental setting and that under IV infection conditions, downregulation of albumin uptake is independent of GSK3β activation.

The contribution of MMP to the pathology of ALI and ARDS has been studied for decades. Pro-inflammatory cytokines induce MMP overexpression and increase activity of these proteases, thereby participating in airway and alveolar remodeling, where MMP play a dual role, driving both destructive pathology and tissue repair. Chemical inhibition of MMP in ALI has been shown to limit lung damage, presenting a potential therapeutic strategy for the treatment of this pathology (47, 48).

According to our data, numerous MMP genes were upregulated in both AEC and BAL fluid immune cells of control and IV-infected mice. We observed a significant upregulation of MMP-19 expression in infected AEC samples. MMP-19, also known as RASI-1, has been reported to utilize ECM components as substrates, suggesting its involvement in tissue remodeling (47). Additionally, research has shown that MMP-19 can protect against the development of fibrosis after lung injury (49).

The upregulation of MMP-14 (also known as MT1-MMP) in BAL fluid cells from infected mice was a striking observation, showing a 56-fold-change. Collagen, gelatin, fibronectin, and laminin, as well as non-ECM proteins such as Pro-MMP-2 and -13, LRP1, and CD44, are among the known MMP-14 substrates in the ECM (40, 47, 50). MMP-14 is a membrane-anchored protease that is regulated by a mechanism in which the active enzyme goes through a series of processing events, either autocatalytic or mediated by other proteases, resulting in the shedding of the catalytic domain. As a result, the proteolytic activity would shift from the plasma membrane to the pericellular environment (reviewed by Hernandez-Barrantes et al.) (51).

Talmi-Frank et al. also found extremely elevated levels of MMP-14 mRNA expression in IV H1N1-infected mice, where the majority of MMP-14-overexpressing cells after 5 days of infection were myeloid immune cells (52). Their study revealed significant morphologic and compositional changes in the ECM of IV-infected lungs, including the depletion of fibrillar collagens. The deleterious effects on the ECM were blocked when mice were treated with an MMP-14 specific inhibitor antibody, but not with the IV inhibitor oseltamivir, indicating that the effect was due to the protease’s activity. Furthermore, when the researchers used a second mouse model of co-infection of IV with *S. pneumoniae*, they found that treatment with the specific MMP-14 inhibitor antibody significantly improved the survival of co-infected mice, compared to the bacteria-only infected control. Untreated mice showed bacteremia and dissemination of *S. pneumoniae* into the spleen and liver, whereas the infection in treated mice remained limited to the lungs. These findings suggest that the damage caused by infiltrating immune cells and their overexpression of MMP-14 contributes to severe influenza infection, highlighting the potential therapeutic strategy of timely MMP-14 inhibition. Since MMP-14 can act on the pericellular environment, the downregulation of megalin expression could also be explained by the contribution of a possible crosstalk between cells, where MMPs produced by immune cells act on alveolar epithelial cells. However, our group has shown that MMP-14 is not the only MMP involved in the proteolysis of megalin (17) and also the inhibition of other proteases could potentially be of use.

The exact mechanism of action of MMP-14 and its regulation by IV infection in the lung has not yet been described. MMP-14 is known to trigger MMP-2 activation in response to bleomycin in AEC (53) and to facilitate extracellular domain proteolysis of LRP1 (40). Our prior research indicated that MMP-2 and MMP-14 directly interact with megalin at the plasma membrane of AEC. This suggests that megalin may scaffold their interaction, activating the enzyme for self-ectodomain shedding (16). NSC405020 inhibits MMP-14 homodimerization via the MMP-14 PEX domain (45) and hence the collagenase activity of the enzyme. MMP-14 was present, although not upregulated, in our *in-vitro* models. Administration of this inhibitor in MLE-12 cells and PCLS cultures partially restored megalin expression and albumin uptake, hinting at the role of MMP-14 in megalin shedding. Our data align with the report of Mazzocchi et al., showing that TGF-β1-treated RLE-6TN cells had increased megalin shedding and MMP-14 expression, while MMP-14 siRNA restored megalin and albumin levels (17). In our studies, however, albumin uptake remained lower in infected cultures even after application of NSC405020 than in controls, suggesting that MMP-14 *per se* only partially drives the reduction of albumin uptake in AEC upon IV exposure, with other factors involved. Thus, nonspecific MMP inhibitors may be potentially useful for ALI/ARDS treatment.

Our study has revealed a significant downregulation of megalin at both the transcriptional and translational levels during IV infection. The question of what triggers this downregulation in AEC remains partially unresolved. It is worth noting that several of the molecules that affect megalin expression at both the mRNA and protein levels are its ligands (54). For example, vitamins A and D, which are endocytosed by megalin, have been shown to increase megalin mRNA in various cell types (55). It has also been shown that albumin concentrations higher than 10 mg/mL promote downregulation of megalin at both mRNA and protein levels and apoptosis after 24 h of treatment in renal proximal tubule cells, an effect that appears to be independent of TGF-β1 activity (56).

Research conducted by Biemesderfer and colleagues has shown that megalin undergoes a Notch-like processing mechanism, where ligand binding activates a signaling pathway that can result in gene regulation. They suggested that RIP of megalin in the kidneys can be induced by vitamin D-binding protein, which may regulate genes related to vitamin D metabolism. However, the authors also noted that it is essential to investigate whether all megalin ligands activate processing equally and whether they activate or repress the same genes (21). Following post-translational modifications, megalin (being a glycoprotein) undergoes heterogeneous glycosylation steps, with the most abundant glycans being SAα2,6Gal and SAα2,3Gal (57). As IV internalization relies on the interaction between its HA protein and cell surface receptors containing SA residues bound to galactose, megalin-mediated endocytosis is likely one of the mechanisms involved in viral entry. Thus, it is possible that the interaction between the virus and megalin may trigger the downregulation of the receptor.

To our knowledge, our study is the first to report a significant decrease in megalin expression and albumin uptake in MLE-12 cells, PCLS, and AEC from mice infected with IV. While we demonstrated the potential therapeutic benefits of MMP inhibition in enhancing protein clearance during ALI, the precise mechanisms that lead to megalin downregulation during IV infection and the full extent of its impact on protein clearance in the lung require further investigation.

## Data availability statement

The datasets presented in this study can be found in online repositories. The names of the repository/repositories and accession number(s) can be found below: GSE241323 (GEO).

## Ethics statement

The animal study was approved by the Animal Ethics Committee of the Regierungspraesidium Giessen, Giessen, Germany. The study was conducted in accordance with the local legislation and institutional requirements.

## Author contributions

Conceptualization, AA-B, and IV. Investigation, AA-B, VK and SG. Methodology, AA-B, VK, CM and SG. Formal analysis, AA-B, VK, CM, SG and IV. Interpretation of data, AA-B, VK, CM, CS, and IV. Resources. CS, SH, and IV. Writing—original draft preparation, AA-B. Writing—review and editing, AA-B, VK, CM, SG, RM, WS, SH, CS, and IV. Visualization, AA-B and VK. Supervision, IV. Project administration, AA-B, and IV. Funding acquisition, WS, CS, and IV. All authors contributed to the article and approved the submitted version.
