# Supplementary material for: Influenza virus decreases albumin uptake and megalin expression in alveolar epithelial cells
Source: Front Immunol. 2023 Sep 1;14:1260973. doi: 10.3389/fimmu.2023.1260973 (PMC10505651; doi:10.3389/fimmu.2023.1260973)
Supplement: Supplementary file 1 [file DataSheet_1.pdf]

Supplementary figure 1

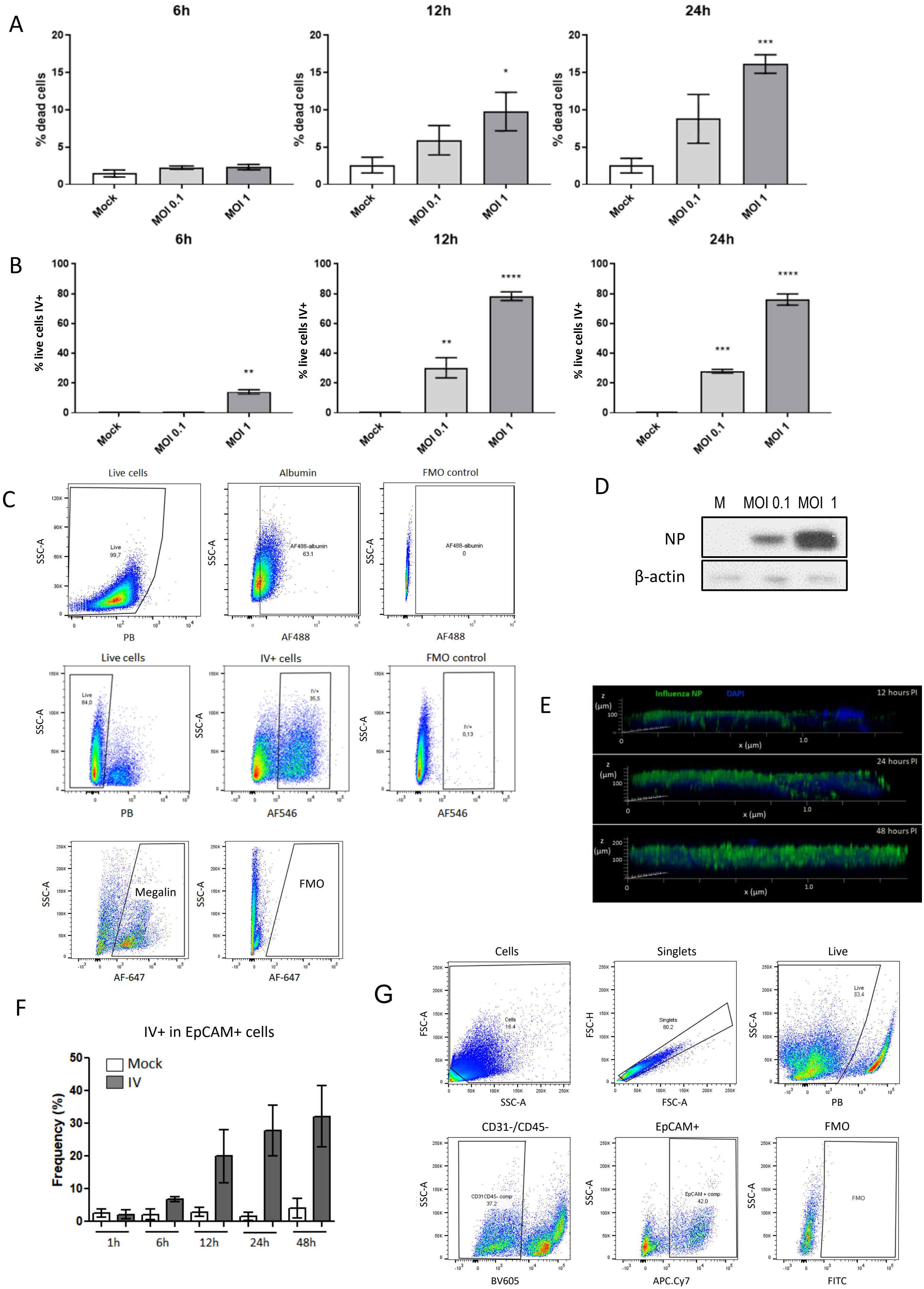

Supplementary figure 1 *Characterization of influenza infection in MLE-12 cells and PCLS.* ***A-C.*** MLE-12 cells were inoculated with PR8 at MOI 0.1 and 1 and analyzed at 6 h, 12 h and 24 h. **A:** Flow cytometric analysis showing cell death percentages for each time point and MOI. **B:** Cells were stained with a specific anti-IV antibody and infection levels assessed as a percentage of live cells. **C:** Gating strategy showing representative dot plots for dead cell exclusion (live cells depicted as PacificBlue-), albumin-AF488 detection, and fluorescence minus one (FMO) control (upper panel), IV infected cells (AF546+) and a representative FMO control for AF546 (IV+, middle panel), and megalin detection and its FMO control (lower panel). **D:** MLE-12 cells were infected with IV MOI 0.1 and MOI 1 for 24 h and western blot analysis of Influenza nucleoprotein (NP) was performed. **E-G:** PCLS were inoculated with IAV and incubated for the shown time periods. **E:** Confocal microscopy lateral view images showing the influenza NP signal (green) and DAPI staining (blue) in PCLS inoculated with PR8 $1x10^6$ pfu per slice, and fixed in PFA 4% after 12 h, 24 h, or 48 h. **F:** FC analysis of influenza proteins (IV) expression in AECs obtained from PCLS single-cell suspensions, 1 h, 6 h, 12h 24h and 48h after inoculation. All bar graphs show mean ± SD of 5 to 6 independent experiments. Statistic comparisons are relative to mock controls. **G:** Representative gating strategy showing dead and CD45+/CD31+ cells exclusion, EpCAM+ cells selection, and the FMO control for IV staining.

Supplementary figure 2

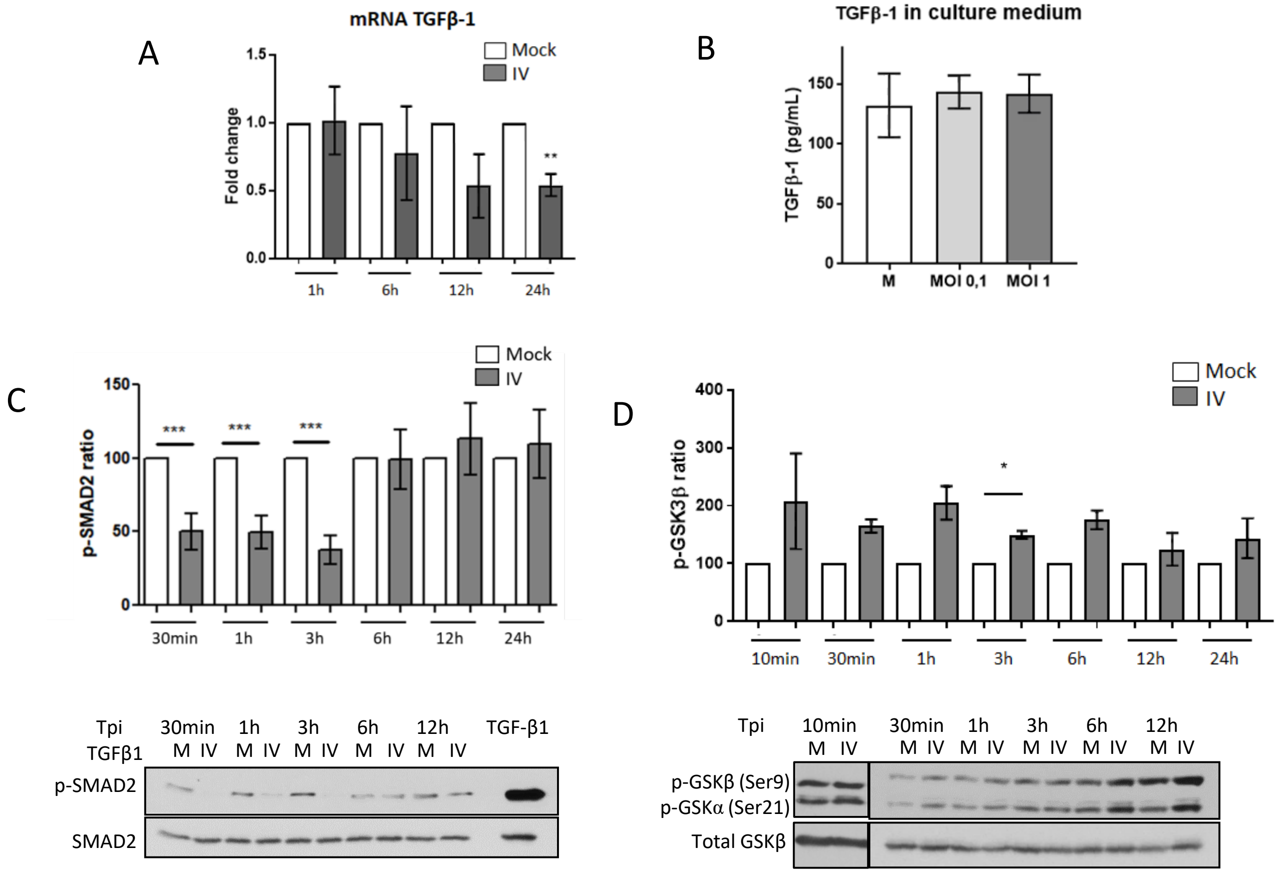

Supplementary figure 2 *Influenza virus infection did not increase TGF-β1 oor GSK3β Ser9 phosphorylation and activation in MLE-12 cells up to 24 h post influenza infection.* **A:** mRNA was extracted from MLE-12 cells lysates, mock and IV-inoculated, at different timepoints, and transcription levels of TGFβ-1 were quantified by qPCR. **B:** ELISA results for active TGF-β1 levels measurement in MLE-12 culture medium 24h p.i. for mock (control), MOI 0.1 and MOI 1. **C:** Densitometry quantification (upper panel) and rrepresentative blots (lower panel) of western blot analysis of phospho-SMAD2 in relation to total protein in MLE-12 cell lysates inoculated with IV at MOI1 (IV) and analyzed at different time points. **D:** Densitometry quantification (upper panel) and rrepresentative blots (lower panel) of western blot analysis of phospho-GSK3β. The antibody used to detect phosphorylation levels in GSK3 is able to bind to GSK3α as well and hence it shows two distinct bands. Tpi = time post infection. TGFβ-1: positive control for SMAD2 phosphorylation, 20 ng/mL, 30 min. All bar graphs show mean ± SD of 3 to 7 independent experiments. Statistic comparisons are relative to mock controls.

Supplementary figure 3

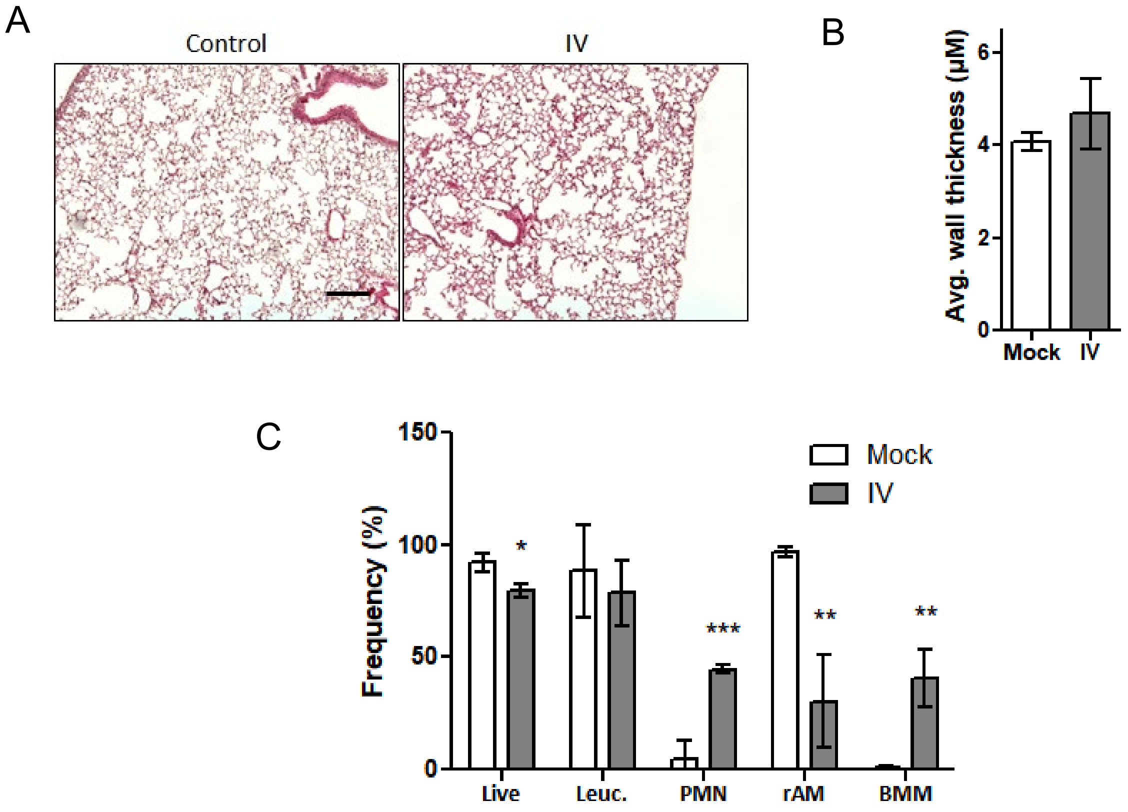

Supplementary figure 3 *IV infection changes in tissue architecture and cell composition in mice lungs 5 days post-inoculation.* **A:** Light micrograph showing a hematoxilin/eosin staining of lung cuts from control and IV-infected mice. Scale bar 100 µm. Zoom 10X. **B:** Average thickness of the alveolar-capillary wall in mock and infected (IV) samples. **C:** BAL fluid cells were analyzed by FC. Graph shows the percentages of live cells (Live), total leucocytes (Leuc.), Polymorphonuclear cells (PMN), tissue-resident alveolar macrophages (TR-AM), and bone marrow-derived macrophages (BMDM).
